# Differential Response to Injury in Fetal and Adolescent Sheep Hearts in the Immediate Post-myocardial Infarction Period

**DOI:** 10.3389/fphys.2019.00208

**Published:** 2019-03-05

**Authors:** Mitchell C. Lock, Jack R. T. Darby, Jia Yin Soo, Doug A. Brooks, Sunthara Rajan Perumal, Joseph B. Selvanayagam, Mike Seed, Christopher K. Macgowan, Enzo R. Porrello, Ross L. Tellam, Janna L. Morrison

**Affiliations:** ^1^Early Origins of Adult Health Research Group, School of Pharmacy and Medical Sciences, University of South Australia, Adelaide, SA, Australia; ^2^Mechanisms in Cell Biology and Disease Research Group, School of Pharmacy and Medical Sciences, University of South Australia, Adelaide, SA, Australia; ^3^Preclinical, Imaging and Research Laboratories, South Australian Health and Medical Research Institute, Adelaide, SA, Australia; ^4^Cardiac Imaging Research Group, Department of Heart Health, South Australian Health and Medical Research Institute, Flinders University, Adelaide, SA, Australia; ^5^The Hospital for Sick Children, Division of Cardiology, Toronto, ON, Canada; ^6^Murdoch Children’s Research Institute, The Royal Children’s Hospital, Parkville, VIC, Australia; ^7^Department of Physiology, School of Biomedical Sciences, University of Melbourne, Parkville, VIC, Australia

**Keywords:** cardiac, fetus, myocardial infarction, proliferation, repair, sheep

## Abstract

**Aim:** Characterizing the response to myocardial infarction (MI) in the regenerative sheep fetus heart compared to the post-natal non-regenerative adolescent heart may reveal key morphological and molecular differences that equate to the response to MI in humans. We hypothesized that the immediate response to injury in (a) infarct compared with sham, and (b) infarct, border, and remote tissue, in the fetal sheep heart would be fundamentally different to the adolescent, allowing for repair after damage.

**Methods:** We used a sheep model of MI induced by ligating the left anterior descending coronary artery. Surgery was performed on fetuses (105 days) and adolescent sheep (6 months). Sheep were randomly separated into MI (*n* = 5) or Sham (*n* = 5) surgery groups at both ages. We used magnetic resonance imaging (MRI), histological/immunohistochemical staining, and qRT-PCR to assess the morphological and molecular differences between the different age groups in response to infarction.

**Results:** Magnetic resonance imaging showed no difference in fetuses for key functional parameters; however there was a significant decrease in left ventricular ejection fraction and cardiac output in the adolescent sheep heart at 3 days post-infarction. There was no significant difference in functional parameters between MRI sessions at Day 0 and Day 3 after surgery. Expression of genes involved in glucose transport and fatty acid metabolism, inflammatory cytokines as well as growth factors and cell cycle regulators remained largely unchanged in the infarcted compared to sham ventricular tissue in the fetus, but were significantly dysregulated in the adolescent sheep. Different cardiac tissue region-specific gene expression profiles were observed between the fetal and adolescent sheep.

**Conclusion:** Fetuses demonstrated a resistance to cardiac damage not observed in the adolescent animals. The manipulation of specific gene expression profiles to a fetal-like state may provide a therapeutic strategy to treat patients following an infarction.

## Introduction

Cardiovascular disease is the most common underlying cause of death in most developed countries (World Health Organization [WHO], 2017). There has been a decrease in mortality rates from CVD over the last 60 years, which is attributed to improvements in the prevention, detection, and management of CVD. However, as the adult human heart has very little capacity to regenerate after damage many patients suffer chronic damage to the heart muscle, resulting in a greater likelihood of heart failure in later years (Australian Institute of Health and Welfare [AIHW], 2015). Current secondary prevention approaches after acute coronary syndromes focus on addressing the ongoing symptoms and risk factor modification to prevent re-infarction (Dalal et al., 2015). The current lack of effective treatments for cardiac repair necessitates the development of new approaches to repair heart damage.

Unlike the adult human heart, the adult zebrafish, newborn mouse, neonatal pig, and fetal sheep, have a remarkable capacity for cardiac regeneration/repair after a MI (Herdrich et al., 2010; Jopling et al., 2010; Porrello et al., 2011; Zhu et al., 2018). This regenerative response has been attributed to the plasticity of immature cardiomyocytes, but the ability for repair is inevitably lost in adult mammals (Herdrich et al., 2010; Jopling et al., 2010; Porrello et al., 2011). While the neonatal mouse has a significant capacity to repair after a MI it is unclear why this regenerative capacity is lost in humans and other large mammals, but it appears to coincide with the timing of heart development in relation to birth and the favorable environment of the developing heart allowing for tissue repair.

A number of key differences exist between humans and rodents/zebrafish models in terms of cardiomyocyte maturation (Lock et al., 2018). Firstly, multinucleation status and ploidy of cardiomyocytes differ between species; for example the adult human heart contains mostly quiescent mononucleated cardiomyocytes with a large proportion of tetraploid (4c) nuclei (Adler, 1991). Other species such as mice, rats, and sheep have mostly binucleated cardiomyocytes in adult life, and seem to lose proliferative capacity in paralell with the mulitnucleation process, which occurs with different developmental timing in these species (Kellerman et al., 1992; Li et al., 1996; Soonpaa et al., 1996; Burrell et al., 2003). Secondly, the timing of cardiomyocyte quiesence differs in relation to oxygen availability, with humans and sheep losing much of their proliferative capacity before birth when PaO_2_ is still relatively low (Soothill et al., 1986; Orgeig et al., 2010; Duan et al., 2017b). Though there is some evidence that human neonatal cardiomyocytes retain some proliferative capacity up to one year after birth, this level of proliferation is much lower than in fetal life (Mollova et al., 2013; Gleadle and Mazzone, 2016). This is in contrast to zebrafish that remain in a low PaO_2_ environment permanantly, as well as mice and rats, which retain proliferative capacity of cardiomyocytes for a period after birth when PaO_2_ is relatively high (Porrello et al., 2011; Puente et al., 2014). Thirdly, thyroid hormone has also been identified as a major modulator of post-natal cardiomyocyte proliferation in mice and rats (Naqvi et al., 2014), however, the major spike in thyroid hormone occurs before birth in large animals at the time when cardiomyocytes are transitioning from proliferative to hypertrophic growth (Thorburn and Hopkins, 1973; Chattergoon et al., 2012; Forhead and Fowden, 2014). The changes in cardiac gene expression in rodents during the perinatal period are difficult to interpret due to confounding changes in cardiac metabolism that occur alongside changes in cardiac development. Given these species differences, it is clear that some biological mechanisms that are important in small animals may not translate into large animals or humans. It is therefore necessary to investigate not only small animals, but also the regenerative capacity of fetuses compared to adults in clinically relevant large animal models, to help identify the critical mechanisms that might improve the response to infarction in humans.

The sheep heart is capable of regeneration after a MI at 65–76 days gestation, but not for the same insult after birth (Herdrich et al., 2010; Zgheib et al., 2014). Characterizing the response to MI in the regenerative sheep fetus compared to the post-natal non-regenerative adolescent heart may reveal key morphological and molecular differences that could equate to the response to MI in humans. We hypothesize that the immediate response to injury in (a) infarct compared with sham, and (b) infarct, border and remote tissue, in the fetal sheep heart will be fundamentally different to the adolescent, allowing for repair after damage. We have therefore investigated the differential responses of cardiac tissue gene expression after MI in the fetus and adolescent sheep heart 3 days after infarction.

## Results

### Decreased Left Ventricular Ejection Fraction and Left Ventricular Cardiac Output in Adolescent Sheep After MI

To confirm the presence of MI after ligation of the LAD, we utilized MR imaging both immediately after surgery and 3 days after infarction with a gadolinium chelate contrast agent: the gold standard in human patients. The size of the infarct in adolescents was 10.9 ± 2.1% of the LV, with no infarct tissue detected in sham animals. Fetal infarct tissue was successfully detected in MI animals, as previously published (Duan et al., 2017b). Ligation of the second diagonal of the LAD was reproducible and resulted in a higher survival rate in adolescent sheep. This method produced an infarct size smaller than previous studies in sheep (∼20% of LV myocardium) (Herdrich et al., 2010; Zgheib et al., 2014), but adequately large for analysis of the molecular response to infarction. There was a significant decrease in LVEF and LVCO 3 days after ligation of the LAD (*P* < 0.05, Table 1). Vascular shunts in the fetal circulation result in a majority of the systemic circulation driven by right ventricular output rather than the LV (Seed et al., 2012). This difference in cardiac function between the fetus and adult, in addition to the much higher heart rate in fetal life, is likely responsible for less functional changes after infarction the fetus.

**Table 1:** MRI measures of cardiovascular function 3 days post-MI.

Outcome Measure	Fetal Sheep		Adolescent Sheep	
	Sham *n* = 5	MI *n* = 5	Sham *n* = 5	MI *n* = 5
Body weight (kg)	1.3 ± 0.4	1.4 ± 0.2	30.5 ± 1.6#	32.3 ± 4.0#
Heart weight (g)	9.8 ± 2.7	11.1 ± 2.1	165.1 ± 13.0#	180.0 ± 30.2#
Left ventricular end diastolic volume (ml)	4.1 ± 0.4	4.5 ± 0.9	86.3 ± 19.9#	107.7 ± 21.9#
Left ventricular end systolic volume (ml)	2.1 ± 0.4	1.9 ± 0.5	39.4 ± 12.7#	79.0 ± 9.2#*
Left ventricular stroke volume (ml)	2.1 ± 0.1	2.6 ± 0.8	46.8 ± 7.3#	28.8 ± 16.7#
Left ventricular ejection fraction (%)	50.5 ± 6.3	57.3 ± 10.9	55.0 ± 4.8#	25.3 ± 11.7#*
Left ventricular cardiac output (ml/min)	313 ± 50	428 ± 120	5356 ± 1592#	2866 ± 1383#*

*Data expressed as mean ± standard deviation, ^#^represents significance for age, ^∗^represents significance for treatment *P* < 0.05 assessed by a 2-way Analysis of variance (ANOVA). Fetal sheep = 105 days gestation, Adolescent sheep = 6 months old.*

### Post-mortem Heart Tissue Morphological Analysis

**FIGURE 1:**
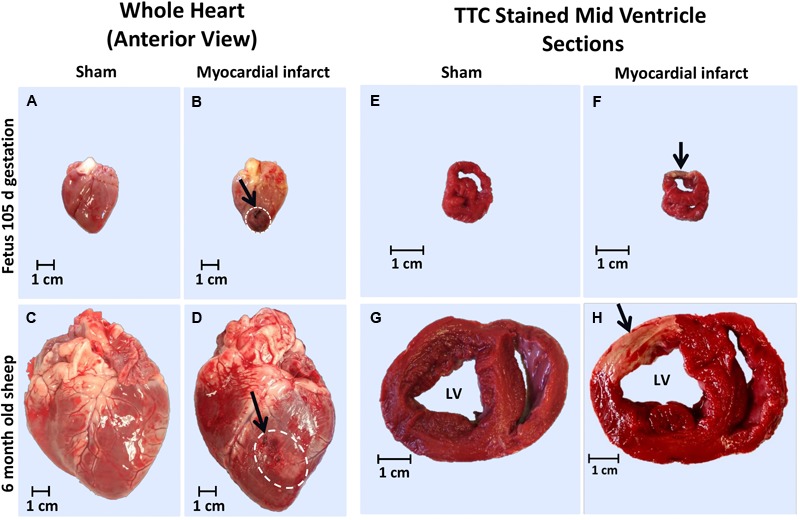
Representative gross heart tissue and TTC stained sections from 105 days fetus and 6 month old adolescent sheep 3 days after MI. Whole hearts at post-mortem of: **(a)** 105 days fetal sham, **(b)** 105 days fetal MI, **(c)** adolescent sham, and **(d)** adolescent MI sheep (arrows and white dashed area indicates ligation site and infarct area respectively); TTC stained heart sections of: **(e)** 105 days fetal sham, **(f)** 105 days fetal MI, **(g)** adolescent sham, and **(h)** adolescent MI sheep (arrow indicates infarct area).

The infarcted fetal heart tissue appeared much darker, more consistent with a bruise, than the classic scarring that was visualized in the adolescent heart tissue (Figure 1). This pathology may indicate some reperfusion of the infarcted tissue in the fetus with the darker coloring consistent with reperfusion type injury, including trapped red blood cells and hemorrhage from ruptured necrotic capillaries. The adolescent hearts were consistent with non-reperfused infarction, with a central area of yellow discoloration surrounded by darker vascularized hyperaemic tissue.

The difference in color of fetal MI tissue allowed for accurate estimation of the epicardial size of the infarct via examination in ImageJ, as previously described (Duan et al., 2017b). The average fetal infarct diameter was 1.24 ± 0.10 cm and was in agreement with the MR imaging (Duan et al., 2017b). As some infarct tissue was collected for molecular analysis and immunohistochemistry, the entire infarct was not sectioned and stained with TTC for total infarct volume. The TTC staining revealed clearly infarcted tissue in both the fetuses and adolescents (Figure 1).

### Increased Collagen Staining in Myocardial Infarct Heart Tissue Compared to Sham Control

**FIGURE 2:**
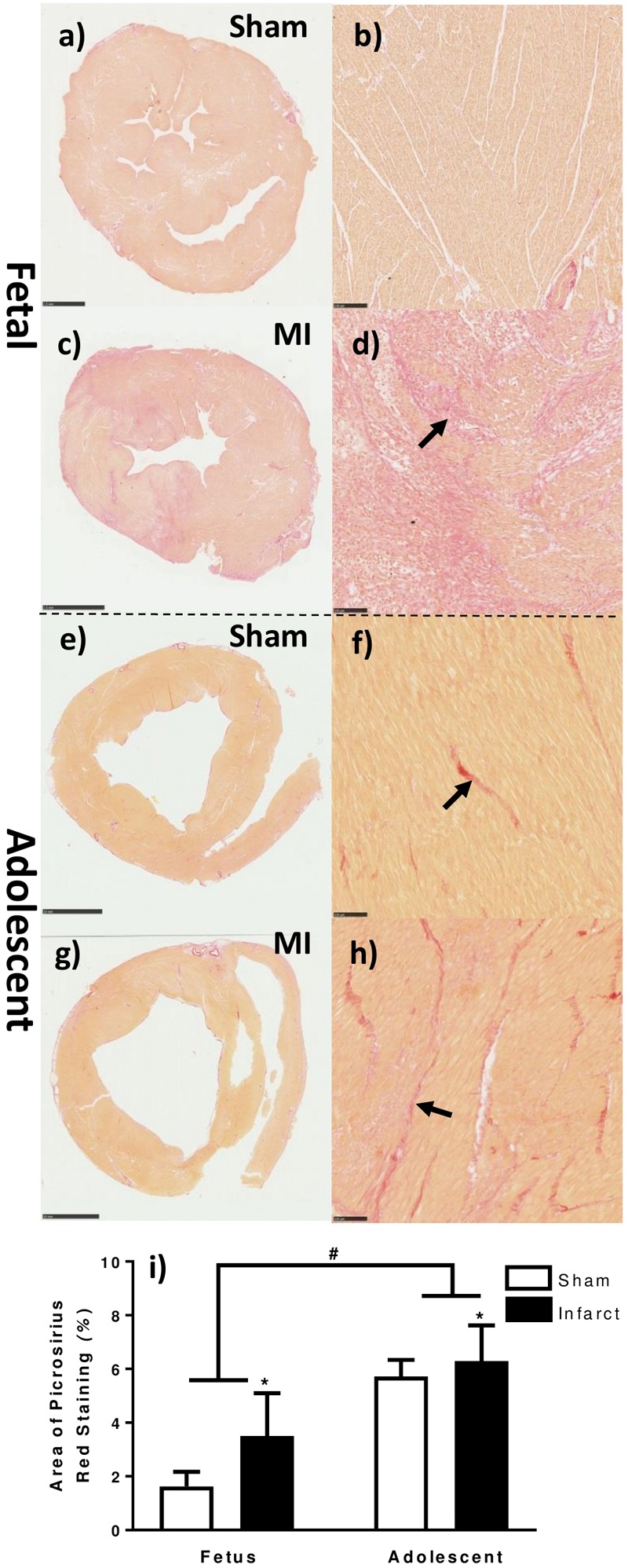
Quantification of collagen staining in the fetal and adolescent heart three days after infarction. **(a)** Whole fetal heart section sham. **(b)** 10× magnification representative micrograph fetal sham. **(c)** Whole fetal heart section MI. **(d)** 10× magnification representative micrograph fetal MI. **(e)** Whole adolescent heart section sham. **(f)** 10× magnification representative micrograph adolescent sham. **(g)** Whole adolescent heart section MI. **(h)** 10× magnification representative micrograph adolescent MI. **(i)** Area of picrosirius red staining in heart tissue sections ± standard deviation. ^∗^represents significant difference as result of treatment (*P < 0.05*). ^#^represents significant difference as a result of age (*P < 0.05*). Analysis was performed using a 2-way ANOVA for treatment group and age (fetuses *n* = 5 per group, adolescent *n* = 5 per group). Scale bars of fetal whole sections = 2.5 mm. Scale bars of adolescent whole sections = 10 mm. Scale bars of micrographs = 100 μm. Arrows demonstrate representative picrosirius red staining.

The area of collagen (Picrosirius Red) staining was increased as a result of age as well as MI (Figure 2). The red collagen staining in the fetal MI animals was mostly distributed around the border zone, consistent with immunohistochemistry staining for macrophages (Figure 2c,d). In the adolescent MI tissue, the red collagen staining was evenly distributed in the infarct and border zone tissue (Figure 2g,h).

### Increased Numerical Density of Ki-67 Positive Cells in Fetal MI Whole-Heart Sections

**FIGURE 3:**
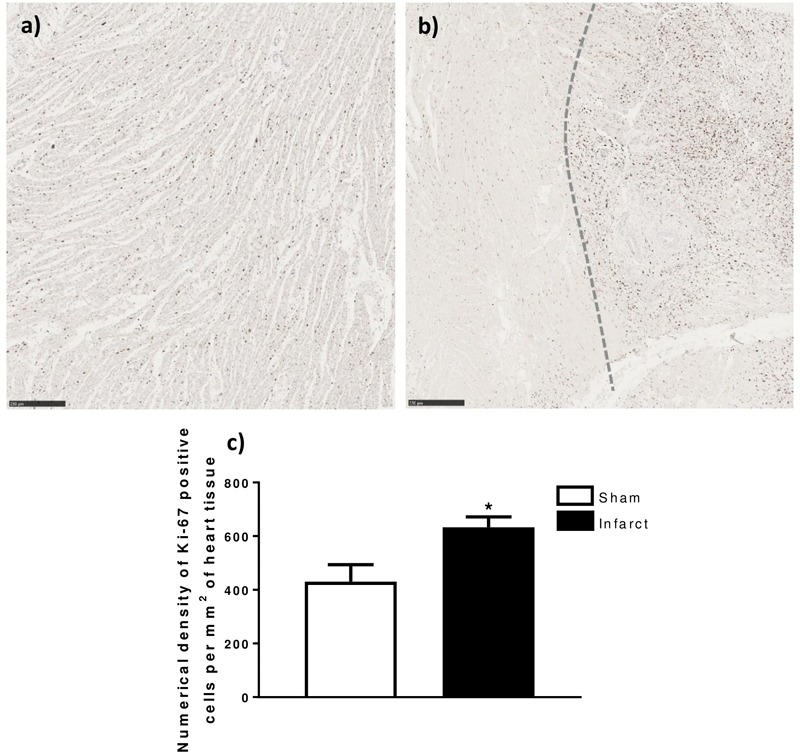
Ki-67 quantification in fetal heart tissue after infarction. **(a)** Representative Ki-67 stained LV heart tissue (5× Magnification) 105 days sham Fetus. **(b)** Representative Ki-67 stained infarct (left side) and border zone (right side) heart tissue (5× Magnificaion) 105 days MI Fetus. **(c)** Numerical density of Ki-67 positive DAB Stained cells per mm^2^ of whole heart tissue sections ± standard deviation. ^∗^represents significant difference as result of treatment (*P < 0.05*). Analysis was performed using a Student’s *t*-test (Sham *n* = 5, Infarct *n* = 5). Scale bars = 250 μm.

**FIGURE 4:**
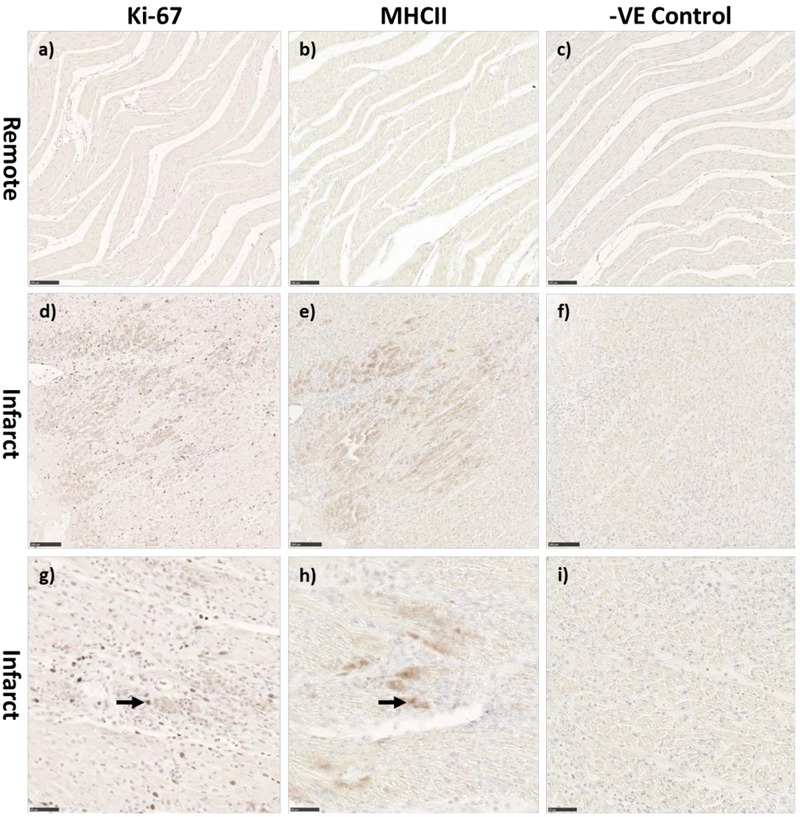
Representative co-localization of Ki-67 and MHC-II staining in the adolescent sheep 3 days after MI. Control remote zone tissue of Ki-67 **(a)** MHCII **(b)** and negative control **(c)** adolescent stained sections (10× Magnification). Infarct area of adolescent Ki-67 **(d)** MHCII **(e)** and negative control **(f)** adolescent stained sections (10× Magnification). Infarct area of adolescent Ki-67 **(g)** MHCII **(h)** and negative control **(i)** adolescent stained sections (20× Magnification). Scale bars 10× magnification = 100 μm, Scale bars 20× magnification = 50 μm. Black arrows represent colocalization of markers.

**FIGURE 5:**
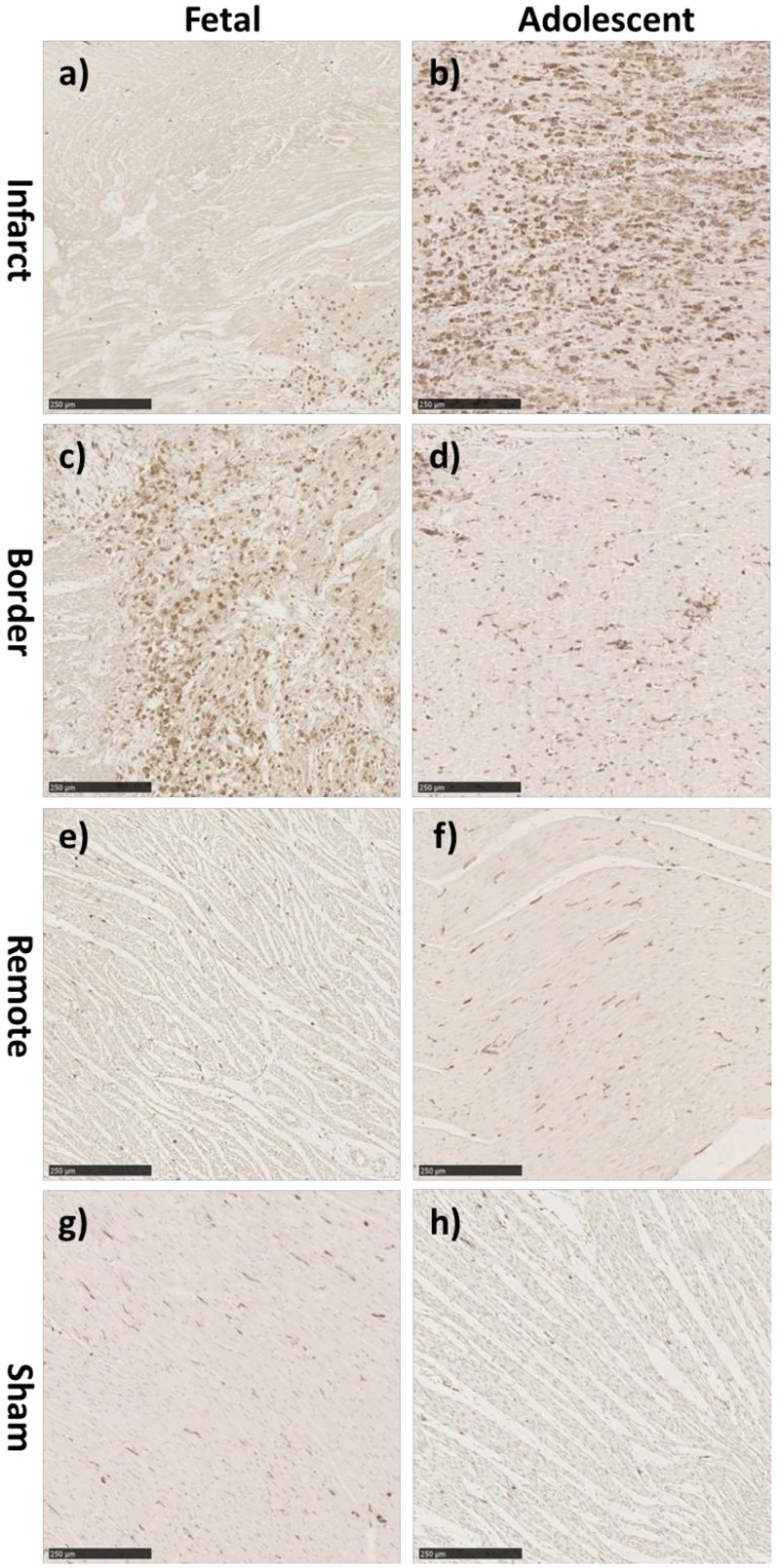
Representative IBA-1 immunohistochemistry staining in the fetus and adolescent sheep LV 3 days post-MI. **(a)** Fetal Infarct tissue, **(c)** fetal border zone tissue, **(e)** fetal remote zone tissue, and **(g)** fetal sham control tissue. **(b)** Adolescent sheep infarct tissue, **(d)** adolescent border zone tissue, **(f)** adolescent remote zone tissue, and **(h)** adolescent sham control tissue (20× Magnification). Scale bars = 250 μm.

There was a significant increase in the numerical density (number of stained cells per mm^2^ of tissue) of Ki-67 stained cells, a marker for cells in the cell-cycle, in fetal whole heart tissue as a result of MI (*P* < 0.05, Figure 3C). In the adolescent sheep, there was no visible Ki-67 staining except in some regions of the infarct area, where the limited number of positive cells were not quantifiable (Figure 4). The staining was clear and distinct in the fetuses, which allowed for accurate quantification of the density of Ki-67 positive cells (Figure 3a). The majority of Ki-67 staining in fetal heart tissue was located in the salvageable border zone tissue adjacent to the MI (Figure 3b). Ki-67 is not a specific marker for proliferation, rather DNA synthesis, and therefore may instead represent multinucleation (Brown and Gatter, 2002; van Amerongen and Engel, 2008). In addition, in adolescent sheep we utilized serial sections of whole heart tissue to differentiate Ki-67 staining of cardiac cells from inflammatory cells by also staining for antigen presenting cell marker MHCII (Figure 4) and macrophage specific marker IBA1 (Figure 5). The MHCII staining demonstrated co-localized staining with the Ki-67 positive areas in the adolescent tissue (Figure 4) and was also consistent with the localisation of IBA1 staining, indicating that the areas of Ki-67 staining in adolescent sheep were most likely due to inflammation and macrophage invasion, rather than cardiac cell proliferation. These data are consistent with the increase in *MKI67* mRNA expression in adolescent sheep infarct compared to border and remote zone (Table 2). The lack of MHCII staining in the fetuses suggests a more limited immune response after MI. Comparing the staining of IBA1 in the fetus with the adolescent sheep, there was a distinct difference in location of the positively stained cells. The adolescent tissue had a large amount of IBA1 positive cell invasion in the infarct area, with the fetuses demonstrating relatively less staining (Figure 5). In comparison, the fetal tissue had the largest proportion of IBA1 positive cells inside the border zone with little staining in the remote zone and sham tissue (Figure 5).

**Table 2:** qRT-PCR mRNA expression of additional genes between Sham, Remote, Border, and Infarct tissue.

Gene	Age	Sham *n* = 5	Remote *n* = 5	Border *n* = 5	Infarct *n* = 5
***Cell cycle regulators***			
*CDKN1A*	Fetus	0.048 ± 0.023	0.049 ± 0.018	0.072 ± 0.045	0.132 ± 0. 0.073*
	Adolescent	0.311 ± 0.060	0.308 ± 0.133a	0.626 ± 0.079b*	0.078 ± 0.041c*
*CDKN1B*	Fetus	0.517 ± 0.035	0.366 ± 0.067a	0.625 ± 0.172b	0.647 ± 0.186b
	Adolescent	0.428 ± 0.141	0.430 ± 0.031x	0.411 ± 0.186xy	0.265 ± 0.069y
***Neuregulin Signaling***			
*ERBB4*	Fetus	0.260 ± 0.037	0.028 ± 0.024a*	0.248 ± 0.088b	0.161 ± 0.057b*
	Adolescent	0.125 ± 0.038	0.128 ± 0.022x	0.081 ± 0.039x	0.012 ± 0.006y*
***Meis1 and Hippo pathway***			
*MEIS1*	Fetus	0.208 ± 0.081	0.159 ± 0.033a	0.310 ± 0.061b*	0.243 ± 0.053ab
	Adolescent	0.166 ± 0.048	0.231 ± 0.144	0.162 ± 0.067	0.085 ± 0.019*
*YAP1*	Fetus	3.289 ± 0.815	2.356 ± 0.172a	5.136 ± 1.018b*	3.997 ± 0.699b
	Adolescent	2.228 ± 0.596	2.206 ± 0.186xy	2.789 ± 0.641y	1.594 ± 0.126x
***Proliferation markers***			
*MKI67*	Fetus	0.119 ± 0.052	0.133 ± 0.012a	0.104 ± 0.019b	0.096 ± 0.015b
	Adolescent	0.008 ± 0.003	0.005 ± 0.002x	0.008 ± 0.009x	0.132 ± 0.030y*
*PCNA*	Fetus	0.124 ± 0.005	0.118 ± 0.014a	0.159 ± 0.022b*	0.185 ± 0.009b*
	Adolescent	0.049 ± 0.012	0.056 ± 0.007x	0.053 ± 0.008x	0.132 ± 0.014y*
***Apoptosis and autophagy***			
*BECN1*	Fetus	0.141 ± 0.019	0.139 ± 0.028	0.127 ± 0.051	0.153 ± 0.027
	Adolescent	0.209 ± 0.038	0.207 ± 0.024x	0.191 ± 0.033xy	0.138 ± 0.015y*
***Hypertrophy***			
*MYH7*	Fetus	4.625 ± 0.299	5.047 ± 2.010a	4.427 ± 0.864a	1.005 ± 0.761b*
	Adolescent	6.011 ± 0.962	7.046 ± 1.165x	8.087 ± 0.869x*	0.044 ± 0.036y*
*RCAN1*	Fetus	0.107 ± 0.008	0.110 ± 0.0316ab	0.081 ± 0.037a	0.137 ± 0.021b
	Adolescent	0.121 ± 0.009	0.197 ± 0.053xy	0.320 ± 0.054x*	0.141 ± 0.053y
*ATP2A2*	Fetus	20.55 ± 5.030	22.27 ± 3.954a	18.18 ± 1.149a	5.289 ± 3.012b*
	Adolescent	30.45 ± 3.882	33.44 ± 7.169x	17.76 ± 7.153x*	1.077 ± 0.299y*
***Fatty acid metabolism***			
*FABP*	Fetus	0.500 ± 0.150	0.604 ± 0.106	0.372 ± 0.165	0.423 ± 0.231
	Adolescent	0.784 ± 0.299	1.335 ± 0.340	1.198 ± 0.248	1.470 ± 0.869*
***Glucose metabolism***			
*PDK4*	Fetus	0.108 ± 0.044	0.030 ± 0.013a	0.093 ± 0.055ab	0.211 ± 0.141b
	Adolescent	1.358 ± 0.793	1.659 ± 0.936x	1.438 ± 1.052x	0.081 ± 0.058y

*Data expressed as mean normalized expression ± standard deviation. Superscript letters (Fetal Sheep; a, b, c and Adolescent Sheep; x, y, z) represent significance between tissue regions (Remote, Border, and Infarct) at each age (*P < 0.05*). ^∗^represents significantly different data from the sham animals at each age (*P < 0.05*). Analyses between tissue regions (Infarct vs. Border vs. Remote) at each age were assessed using a nested Analysis of variance (ANOVA). A Bonferroni *post hoc* test was performed with multiple comparisons for each tissue region against the Sham tissue. Fetal sheep = 105 days gestation, Adolescent sheep = 6 months old.*

### Effect of Development and MI on Cardiac Gene Expression

#### Growth Factors and Cell Cycle Regulators

**FIGURE 6:**
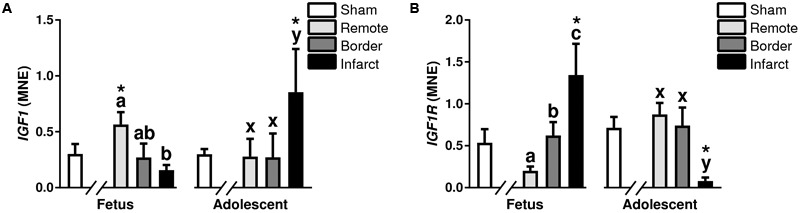
Gene expression of Growth Factor *IGF* pathway in sheep 3 days post-MI. Mean normalized expression (MNE) of *IGF1* **(A)** and *IGF1R* **(B)** in Sham, Remote, Border zone, and Infarct Tissue. Superscript letters (Fetal Sheep; a, b, c and Adolescent Sheep; x, y, z) represent significance between tissue regions (Remote, Border, and Infarct) at each age (*P* < 0.05). ^∗^represents significantly different data from the sham animals at each age (*P < 0.05*). Analyses between tissue regions (Infarct vs. Border vs. Remote) at each age were assessed using a nested Analysis of variance (ANOVA). A Bonferroni *post hoc* test was performed with multiple comparisons for each tissue region against the Sham tissue. *n* = 5 per treatment group per age.

There was a decrease in *IGF1* expression in the infarct area compared to the remote zone in fetuses (*P* < 0.05, Figure 6A), and an increase in *IGF1* expression in the adolescent infarct area compared to both the border and remote zones (*P* < 0.05, Figure 6A). The expression of *IGF1* was increased in the fetal remote zone compared to fetal sham tissue, there was also an increase in the infarct tissue compared with sham tissue in adolescent animals (*P* < 0.05, Figure 6A). There was an increase in expression of *IGF1R* in the fetal border and infarcted areas compared to remote zone (*P* < 0.05, Figure 6B), whereas in the adolescent sheep, there was a decrease in *IGF1R* in the infarct area compared to the border and remote zones (*P* < 0.05, Figure 6B). *IGF1R* was increased in the fetal infarct area compared to sham tissue, and downregulated in the adolescent infarct tissue compared with sham tissue (*P* < 0.05, Figure 6B).

The expression of *CDKN1A (p21)*, encoding a regulator of cell cycle progression at G1 and S phase (Brooks et al., 1998), was increased in the fetal infarct tissue compared to sham tissue, and decreased in the adolescent border and infarct area compared to the sham tissue (*P* < 0.05, Table 2). In the adolescent MI hearts, there was an increase in *CDKN1A* mRNA expression in the border zone compared with the remote zone (*P* < 0.05, Table 2), in addition to a decrease in the infarct area compared with the border and remote zone tissue (*P* < 0.05, Table 2). The expression of *CDKN1B* (*p27)*, another cell cycle inhibitor protein was increased in the fetal border and infarct area of compared with remote zone (*P* < 0.05, Table 2). In the adolescent sheep, there was a decrease in expression of *CDKN1B* in the infarct area compared to remote zone (*P* < 0.05, Table 2).

#### Neuregulin Signaling

**FIGURE 7:**
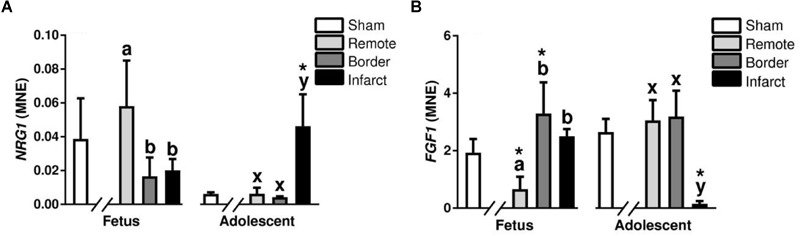
Gene expression of developmental and growth signaling *NRG1* and *FGF1* in sheep 3 days post-MI: Mean normalized expression (MNE) of *NRG1* **(A)** and *FGF1* **(B)** in Sham, Remote, Border, and Infarct Tissue. Superscript letters (Fetal Sheep; a, b, c and Adolescent Sheep; x, y, z) represent significance between tissue regions (Remote, Border, and Infarct) at each age (*P* < 0.05). ^∗^represents significantly different data from the sham animals at each age (*P < 0.05*). Analyses between tissue regions (Infarct vs. Border vs. Remote) at each age were assessed using a nested Analysis of variance (ANOVA). A Bonferroni *post hoc* test was performed with multiple comparisons for each tissue region against the Sham tissue. *n* = 5 per treatment group per age.

Neuregulin 1 is a cardioactive growth factor released from endothelial cells, and is necessary for cardiac development, structural maintenance, and functional integrity of the heart (Lock et al., 2018). There was a decrease in expression of *NRG1* in the fetal border and infarct area compared to remote zone (*P* < 0.05, Figure 7A). In the adolescent sheep, there was an increase in *NRG1* expression in the infarct area compared to border and remote zone (*P* < 0.05, Figure 7A). *NRG1* expression was unchanged in fetal infarct tissue compared to sham tissue, however, there was an increase in the adolescent infarct tissue compared with sham (*P* < 0.05, Figure 7A). *ERBB4* is a gene encoding a subunit of the neuregulin receptor (Lock et al., 2018). *ERBB4* expression was upregulated in the fetal border and infarct area compared to the remote zone (*P* < 0.05, Table 2). *ERBB4* was a downregulated in the adolescent infarct area compared to border and remote zones (*P* < 0.05, Table 2). *ERBB4* was downregulated in the fetal remote and infarct tissue compared to sham tissue (*P* < 0.05, Table 2). *ERBB4* was also downregulated in the adolescent infarct tissue compared to sham tissue.

#### Meis1, Hippo, and FGF Pathway

Meis Homeobox 1 is encoded by the *MEIS1* gene and has a role in normal cardiac development as well as suppression of cardiomyocyte proliferation (Lock et al., 2018). *MEIS1* expression was upregulated in the fetal border tissue compared to sham tissue and decreased in the adolescent infarct tissue compared with sham (*P* < 0.05, Table 2). There was an increase in *MEIS1* expression in the fetal border zone compared with remote zone (*P* < 0.05, Table 2). YAP1 is a transcriptional regulator in the Hippo signaling pathway involved in cellular proliferation and suppressing apoptotic genes (Lock et al., 2018). There was an increase in *YAP1* expression in the fetal border and infarct area compared to remote zone (*P* < 0.05, Table 2). In the adolescent animals, there was a decrease in expression of *YAP1* in the infarct area compared with the border zone (*P* < 0.05, Table 2). *YAP1* was upregulated in the fetal border tissue compared with sham tissue (*P* < 0.05, Table 2). FGF1 is a growth factor and signaling protein that is involved in a broad array of biological processes (Lock et al., 2018). *FGF1* expression was increased in the fetal border and infarct area compared with the remote zone (*P* < 0.05, Figure 7B). In the adolescent sheep, there was a decrease in *FGF1* the infarct area compared to the border and remote zone (*P* < 0.05, Figure 7B). *FGF1* expression was decreased in the fetal remote tissue and increased in the fetal border tissue compared to sham tissue (*P* < 0.05, Figure 7B). *FGF1* was decreased in the adolescent infarct tissue compared with sham tissue (*P* < 0.05, Figure 7B).

#### Cell Proliferation Markers

Marker of proliferation Ki-67 and *PCNA* are markers of cell cycle and proliferation (Arbustini et al., 1993). *MKI67* was decreased in the fetal border and infarct area compared with remote zone but upregulated in the adolescent infarct compared to the border and remote zone (*P* < 0.05, Table 2). *MKI67* was upregulated in the in the adolescent infarct tissue compared to sham tissue (*P* < 0.05, Table 2). *PCNA* expression increased in the fetal border and infarct compared to the remote zone (*P* < 0.05, Table 2). Similarly, the adolescent sheep also had increased *PCNA* expression in the infarct area compared with the border and remote zone (*P* < 0.05, Table 2). *PCNA* was increased in the fetal border and infarct tissue compared to sham tissue, there was also an increase in *PCNA* in the adolescent infarct compared to sham tissue (*P* < 0.05, Table 2).

#### Inflammatory Cytokines

**FIGURE 8:**
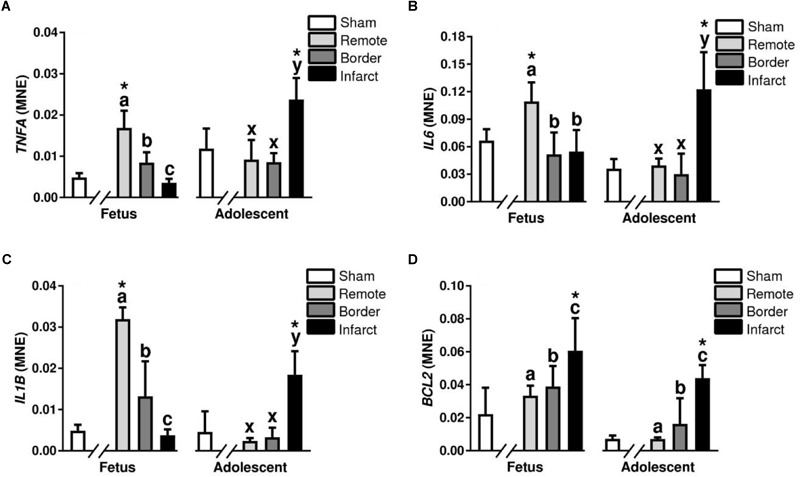
Gene expression of inflammatory cytokines in sheep 3 days post-MI. Mean normalized expression (MNE) of *TNFA* **(A)**, *IL6* **(B)**, *IL1β* **(C)** and *BCL2* **(D)** in Sham, Remote, Border, and Infarct Tissue. Superscript letters (Fetal Sheep; a, b, c and Adolescent Sheep; x, y, z) represent significance between tissue regions (Remote, Border, and Infarct) at each age (*P* < 0.05). ^∗^represents significantly different data from the sham animals at each age (*P < 0.05*). Analyses between tissue regions (Infarct vs. Border vs. Remote) at each age were assessed using a nested Analysis of variance (ANOVA). A Bonferroni *post hoc* test was performed with multiple comparisons for each tissue region against the Sham tissue. *n* = 5 per treatment group per age.

Tumor necrosis factor alpha, *IL6*, and *IL1β* are all pro-inflammatory cytokines responsible for promoting an immune response after infarction (Nian et al., 2004). *TNFA, IL6*, and *IL1β* expression was decreased in the fetal infarct area compared to remote zone (*P* < 0.05, Figure 8). In the adolescent sheep, there was an increase in *TNFA, IL6*, and *IL1β* expression in the infarct area compared to border and remote zones (*P* < 0.05, Figure 8). The expression of *TNFA, IL6*, and *IL1β* was increased in the fetal remote tissue compared to sham tissue and increased in the adolescent infarct compared to sham tissue (*P* < 0.05, Figure 8).

#### Apoptosis and Autophagy

B-cell lymphoma 2 (BCL2) is a mitochondrial membrane protein, that plays an important role in promoting cellular survival and inhibiting the actions of pro-apoptotic proteins (Maejima et al., 2016). There was an increase in the mRNA expression of *BCL2* in the border and infarct for both fetuses and adolescent sheep (*P* < 0.05, Figure 8D). *BCL2* expression was also increased in the infarct tissue compared to sham tissue at both ages (*P* < 0.05, Figure 8D). *BECN1* is a regulator of autophagy through the PI3K pathway (Maejima et al., 2016). In the adolescent sheep *BECN1* expression was downregulated in the infarct compared to the remote zone (*P* < 0.05, Table 2). *BECN1* was also downregulated in the adolescent infarct tissue compared with sham tissue (*P* < 0.05, Table 2).

#### Hypertrophy Markers

**FIGURE 9:**
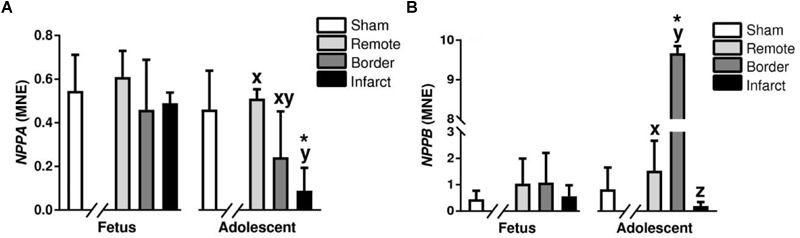
Gene expression of hypertrophy markers *NPPA* and *NPPB* in sheep 3 days post-MI: Mean normalized expression (MNE) of *NPPA* **(A)** and *NPPB* **(B)** in Sham, and Remote, Border, and Infarct Tissue. Superscript letters (Fetal Sheep; a, b, c and Adolescent Sheep; x, y, z) represent significance between tissue regions (Remote, Border, and Infarct) at each age (*P* < 0.05). ^∗^represents significantly different data from the sham animals at each age (*P < 0.05*). Analyses between tissue regions (Infarct vs. Border vs. Remote) at each age were assessed using a nested Analysis of variance (ANOVA). A Bonferroni *post hoc* test was performed with multiple comparisons for each tissue region against the Sham tissue. *n* = 5 per treatment group per age.

Atrial natriuretic peptide (*NPPA*) and Brain natriuretic peptide (*NPPB*) are hormones synthesized in the heart that act to inhibit maladaptive cardiac hypertrophy (Langenickel et al., 2000). *NPPA* was significantly downregulated in the adolescent infarct area compared to remote zone (*P < 0.05*, Figure 9A). The expression of *NPPA* was a significantly decreased in the adolescent infarct tissue compared to sham tissue (*P < 0.05*, Figure 9). *NPPB* expression in the adolescent animals was upregulated in border zone and downregulated in the infarct area compared to the remote zone (*P < 0.05*, Figure 9B). *NPPB* expression was upregulated in the adolescent border tissue compared to sham tissue (*P < 0.05*, Figure 9B). RCAN1 is a cardioprotective protein encoded for by the *RCAN1* gene (Rotter et al., 2014). *RCAN1* expression was increased in the fetal infarct compared to border zone, and decreased in the adolescent infarct area compared to border zone (*P < 0.05*, Table 2). *RCAN1* was also upregulated in the adolescent border tissue compared to sham tissue (*P < 0.05*, Table 2). MYH7 otherwise known as myosin heavy chain beta is expressed in cardiac muscle as a major protein in heart contraction (England and Loughna, 2013). ATP2A2 is an intracellular pump protein involved in the translocation of calcium from the cytosol to the sarcoplasmic reticulum lumen leading to muscular excitation and contraction (Talukder et al., 2009). *MYH7* and *ATP2A2* expression was also decreased in the infarct area compared to remote and border zone in both the fetuses and adolescent animals (*P < 0.05*, Table 2). *MYH7* and *ATP2A2* expression was decreased in the infarct tissue compared to sham tissue in both the fetuses and the adolescent animals (*P < 0.05*, Table 2).

#### Collagens

**FIGURE 10:**
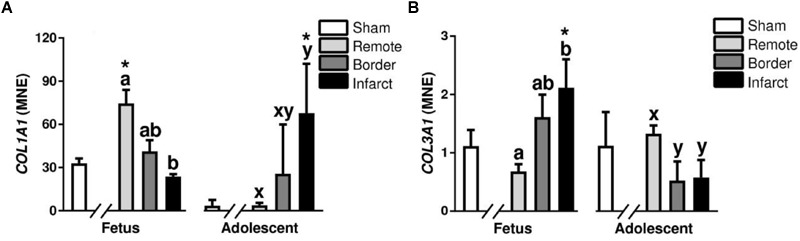
Gene expression of Collagens in sheep 3 days post-MI. Mean normalized expression (MNE) of *COL1A1* **(A)** and *COL3A1* **(B)** in Sham, Remote, Border zone, and Infarct Tissue. Superscript letters (Fetal Sheep; a, b, c and Adolescent Sheep; x, y, z) represent significance between tissue regions (Remote, Border, and Infarct) at each age (*P* < 0.05). ^∗^represents significantly different data from the sham animals at each age (*P < 0.05*). Analyses between tissue regions (Infarct vs. Border vs. Remote) at each age were assessed using a nested Analysis of variance (ANOVA). A Bonferroni *post hoc* test was performed with multiple comparisons for each tissue region against the Sham tissue. *n* = 5 per treatment group per age.

Fibrillar collagen types I and III are the predominant components of cardiac extra cellular matrix (Collier et al., 2012). In the fetal MI hearts, there was a decrease in *COL1A1* in the infarct compared with remote zone (*P* < 0.05, Figure 10A). In the adolescent animals, *COL1A1* was increased in the infarct area compared with the remote zone (*P* < 0.05, Figure 10A). *COL1A1* expression was increased in the fetal remote tissue compared to sham tissue, there was also an increase in expression in the infarcted adolescent tissue compared with sham adolescent (*P* < 0.05, Figure 10A). There was an increase in the expression of *COL3A1* in the fetal infarct compared with remote zone (*P* < 0.05, Figure 10B). In the MI adolescents, there was a decrease in expression of *COL3A1* in both the border and infarct zone compared with the remote zone (*P* < 0.05, Figure 10B). *COL3A1* mRNA expression was increased in the fetal infarct tissue compared to sham tissue (*P* < 0.05, Figure 10B).

#### Fatty Acid Metabolism

**FIGURE 11:**
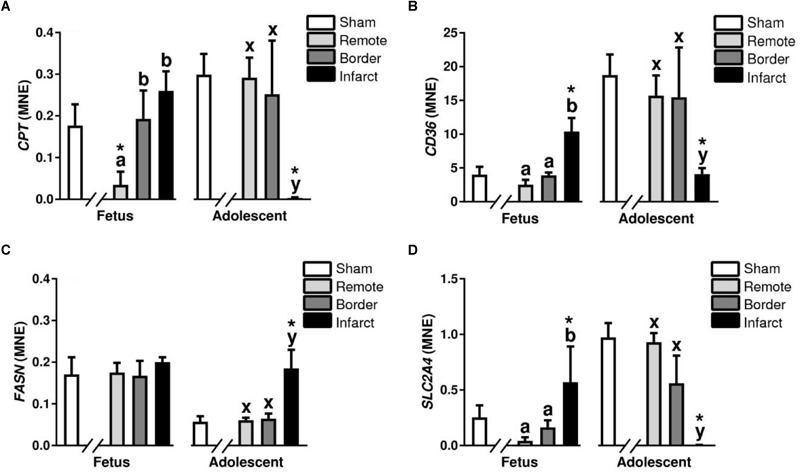
Fatty acid metabolism and glucose transport gene expression in sheep 3 days post-MI. Mean normalized expression (MNE) of *CPT* **(A)**, *CD36* **(B)**, *FASN* **(C)**, and *SLC2A4* **(D)** in Sham, Remote, Border zone, and Infarct Tissue. Superscript letters (Fetal Sheep; a, b, c and Adolescent Sheep; x, y, z) represent significance between tissue regions (Remote, Border, and Infarct) at each age (*P* < 0.05). ^∗^represents significantly different data from the sham animals at each age (*P < 0.05*). Analyses between tissue regions (Infarct vs. Border vs. Remote) at each age were assessed using a nested Analysis of variance (ANOVA). A Bonferroni *post hoc* test was performed with multiple comparisons for each tissue region against the Sham tissue. *n* = 5 per treatment group per age.

Carnitine Palmitoyltransferase (*CPT)* is a key enzyme in the carnitine-dependent transport across the mitochondrial inner membrane and its deficiency results in a decreased rate of fatty acid beta-oxidation (Pauly et al., 1991). In the MI fetuses, there was an increase in expression of *CPT* in the fetal border and infarct areas compared with the remote zone (*P* < 0.05, Figure 11A). The adolescent sheep had decreased *CPT* expression in the infarct area compared with the border and remote zones (*P* < 0.05, Figure 11A). *CPT1* expression was decreased in the fetal remote tissue compared to sham tissue and decreased in the adolescent infarct tissue compared to sham tissue (*P* < 0.05, Figure 11A). CD36 also known as “fatty acid translocase” imports fatty acids into cells and is a member of the class B scavenger receptor family of cell surface proteins (Heather et al., 2006). In the MI fetuses, there was an increase in *CD36* expression in the infarct area compared to border and remote zone (*P* < 0.05, Figure 11B). The MI adolescent animals had decreased expression of *CD36* in the infarct area compared with the border and remote zones (*P* < 0.05, Figure 11B). *CD36* expression was increased in the fetal infarct tissue compared to sham tissue and decreased in the adolescent infarct tissue compared to sham tissue (*P* < 0.05, Figure 11B).

Fatty acid synthase is a multi-enzyme protein encoded by the *FASN* gene which main function is to catalyze the synthesis of palmitate from acetyl-CoA and malonyl-CoA, in the presence of NADPH (Razani et al., 2011). An increase in *FASN* expression was observed in the adolescent infarct area compared to the border and remote zone (*P* < 0.05, Figure 11C). There was also an increase in *FASN* in the adolescent infarct tissue compared to sham tissue (*P* < 0.05, Figure 11C).

#### Glucose Transport and Metabolism

Solute carrier family 2 member 4 is a gene encoding a protein that is responsible for the majority of glucose transport after birth in cardiac tissue (Shao and Tian, 2015). In the MI fetuses, there was an upregulation of *SLC2A4* expression in the fetal infarct area compared to the border and remote zone (*P* < 0.05, Figure 11D). In the adolescent sheep, there was a downregulation of *SLC2A4* in the infarct area compared to border and remote zone (*P* < 0.05, Figure 11D). SLC2A4 was upregulated in the fetal infarct tissue compared to sham tissue, and decreased in the adolescent infarct tissue compared to sham tissue. *PDK4* is a mitrochondrial protein that inhibits the pyruvate dehydrogenase complex, thereby contributing to the regulation of glucose metabolism (Liepinsh et al., 2014). *PDK4* was increased in the fetal infarct area compared to remote zone and downregulated in the adolescent sheep infarct area compared to border and remote zone (*P* < 0.05, Table 2).

## Discussion

### Post-mortem Tissue Observations

The adolescent sheep hearts demonstrated the expected classic scarring response, while the fetal infarcted hearts appeared to have bruised tissue rather than ischemic injury, clearly infarcted tissue only became visible after TTC staining. This response in the fetal tissue may be consistent with previous data in neonatal rodent models that indicates a relatively rapid revascularization of the damaged tissue in response to infarction allowing for some reperfusion of the damaged tissue (Porrello et al., 2013; Aurora et al., 2014). TTC staining of the infarcted tissue demonstrated the expected coloration, with most of the infarcted area remaining unstained in both the fetus and adolescent sheep. The fetal infarcts generally demonstrated a thin segment of TTC staining in the epicardial/pericardial wall, indicating less non-viable tissue than the adolescent sheep. Taken together this morphological response reflects less tissue damage in the fetal heart, setting an environment capable of cardiac repair, compared to the adolescent heart that was consistent with myocardial scarring, and may lead to reduced contractile function.

### Differing Patterns of Cell Cycle and Immune Cell Infiltration After MI in Fetal and Adolescent Sheep

As previously described in the fetal sheep model and neonatal mouse models of MI, cardiac cell proliferation is seen as early as 3 days post-infarction (Herdrich et al., 2010; Porrello et al., 2011; Quaife-Ryan et al., 2017). Immunohistochemistry staining of whole heart tissue sections revealed differing patterns of staining of the cell cycle marker Ki-67 in the fetal infarcted heart tissue (Figure 3a,b) compared with adolescent infarcted heart tissue. This staining was consistent with *MKI67* and *PCNA* qRT-PCR data. The Ki67 positive cells present in the adolescent infarct area were unexpected, however, further analysis demonstrated co-localization of antigen presenting cells and macrophages with the Ki-67 staining in the infarct area. These data indicate that the Ki-67 positive cells within cell cycle in the infarcted area were infiltrating or resident immune cells rather than resident cardiomyocytes or cardiac fibroblasts. Although cardiac fibroblasts are proliferative after infarction, they are generally in the inflammatory and early proliferative phase of the wound healing process, which is unlikely to change the contributing cell types substantially at 3 days post-MI (Talman and Ruskoaho, 2016; Ma et al., 2017). Our inflammatory gene qRT-PCR data also supports the inflammatory phase of post-infarct fibroblast phenotype. However, the differential expression of collagen and inflammatory cytokines in the fetal and adolescent sheep may indicate an accelerated myofibroblast phenotype in the fetuses.

Interestingly, the patterns of IBA-1 staining in the fetus and adolescent sheep were dissimilar. The highest density of macrophages was present in the infarct area of the adolescent sheep, rather than the border zone as seen in the fetal infarcted hearts. The IBA-1 staining was consistent with the Ki-67 staining seen in the fetuses. This result was similar to recent research using the mouse model, which demonstrated increased leukocytes after infarction in both neonatal (regenerative) and adult (quiescent) hearts (Quaife-Ryan et al., 2017). Although Ki-67 is a robust marker of cell cycle, it is not a specific marker for proliferation, and therefore it may also be staining cells undergoing multi-nucleation, rather than proliferation or mitosis (i.e., karyokinesis in the absence of cytokinesis) (Brown and Gatter, 2002; van Amerongen and Engel, 2008). The immediate immune response and localisation of invading inflammatory cells were strikingly different between the adolescent and fetal sheep, it is therefore likely that a coordinated early-inflammatory response is important for successful repair after infarction. Further investigation and identification of the proliferating cells within the infarcted heart may help elucidate the similarities and differences between the fetal and adolescent phenotypes.

### Diminished Pro-inflammatory Cytokines in the Fetal Infarct

Pro-inflammatory cytokine expression is important after infarction to orchestrate the clearance of dead cells and formation of scar tissue, preventing ventricular rupture (Sattler and Rosenthal, 2016). There was a similar expression profile for all pro-inflammatory cytokines after infarction in adolescent sheep, which consisted of an upregulation of these genes after infarction. This response was consistent with the literature describing immunohistochemistry staining of CD45 (Zgheib et al., 2014; Han et al., 2015), a broad marker expressed on a large number of leukocytes, and matched the invasion of macrophages observed with IBA1 staining. The increased expression of pro-inflammatory cytokines in the fetal remote area could be associated with the increased load on the non-damaged myocardium, as seen in models of pressure overload (Shioi et al., 1997; Sun et al., 2007; Souders et al., 2012). The majority of expression of these pro-inflammatory cytokines is likely due to invading leukocytes including macrophages, which in turn increase the production of extracellular matrix from cardiac fibroblasts. Interestingly, IL6 has multiple roles in cardiac and skeletal muscle tissue, promoting cardiomyocyte proliferation following neonatal mouse cardiac injury (Han et al., 2015) and also acting as a potent myokine with a mostly anti-inflammatory role (Muñoz-Cánoves et al., 2013). In skeletal muscle, this myokine is elevated after exercise and, although IL6 has previously been classified as a pro-inflammatory cytokine, its elevation is not associated with muscle damage, but rather physiological hypertrophy (Muñoz-Cánoves et al., 2013). The increases in *IL6* expression may be indicative of macrophage expression rather than myocyte expression, resulting in a pro-inflammatory response through NFκB activation. This was different to the response seen by Zgheib et al. (2014) and may reflect the different age of the fetuses used in each study (a more mature immune system in the fetuses used in our study). There is evidence that distinct macrophage populations derived from embryonic rather than adult-monocyte lineages are required for regeneration in the neonatal mouse through paracrine effects that drive angiogenesis and cardiomyocyte proliferation following injury (Aurora et al., 2014; Lavine et al., 2014). Regardless of the role of IL6 in the heart after infarction, it is clear that a large pro-inflammatory response is occurring in the adolescent animals, as seen with the upregulation of *TNFA* and *IL1β*, which was absent or suppressed in the fetuses. This response in fetuses can be expected given the largely anti-inflammatory environment that the fetus inhabits throughout gestation [due to high expression of anti-inflammatory cytokines IL-4 and IL-10 (Chatterjee et al., 2014)] and may contribute to the increased regenerative capacity of the heart during fetal life.

### Regulation of Apoptosis and Autophagy and Hypertrophy After Infarction

Apoptosis and autophagy are tightly controlled processes within the heart, allowing for extensive cardiac remodeling after birth in the absence of cardiomyocyte proliferation (Kajstura et al., 1995; Smolich, 1995). The changes in *BECN1* expression may indicate a possible resistance to stress induced autophagy within the fetuses, however, further analysis of this pathway is required to elucidate this complex process. In addition, the unchanged *NPPA* and *NPPB* expression in the fetal heart may indicate a resistance to apoptosis and hypertrophic signaling. The fetal heart, however, is still undergoing changes to cardiomyocyte endowment in late gestation, including cardiomyocyte hypertrophy and cardiomyocyte apoptosis, a process that is mostly downregulated after birth. The expression of apoptotic and hypertrophic markers may therefore be overshadowed by developmental changes (Anversa et al., 1986). The expression profile of hypertrophic markers in the adolescent animals makes sense physiologically given that the infarct area will form scar tissue, with the surrounding tissue required to increase its hemodynamic load to retain normal cardiac function. The expression pattern of apoptotic markers may indicate that the fetal heart is downregulating apoptosis in distal areas of the infarcted myocardium in favor of replacing damaged cardiomyocytes in the infarcted area, through apoptosis of damaged cells and proliferation of healthy tissue in the nearby border zone.

### Differing Patterns of Collagen Deposition Between Fetus and Adolescent Sheep

Extracellular matrix deposition is known to be important for the immediate response after ischemic cardiac injury in both zebrafish and mice allowing for repair (Chen et al., 2016; Bassat et al., 2017). In our model, there was an increase in the percentage of staining of picrosirius red in infarcted animals compared with sham animals in both age groups. This difference occurred in addition to an increase in staining with age, reflecting the known increase in fibrosis of cardiac tissue with age (Biernacka and Frangogiannis, 2011). The changes in collagen after infarction were somewhat unexpected at 3 days post-infarction, especially in the fetus where there was a large increase over the sham animals but no change in cardiac function. The pattern of immunohistochemistry staining for mature macrophages closely matched the collagen staining. This result is logical given that macrophages stimulate the production of pro-inflammatory cytokines such as 1L-1β and TNF-α, which upregulate and activate matrix metalloproteinases (MMPs) that are initially responsible for collagen degradation and, subsequently, collagen matrix deposition (Nian et al., 2004). Interestingly, the distribution of collagen staining in the fetuses was localized mostly around the border rather than in the infarct area, whereas in the adolescent animals the staining was mostly observed in the infarct area with less in the border zone. *COL1A1* is the major component of type 1 collagen forming a large portion of the extracellular matrix (Pauschinger et al., 1998). Interestingly, there were opposite expression profiles between fetuses and adolescent sheep for both *COL1A1* and *COL3A1* between the individual tissue regions, indicating a reciprocal relationship between the two collagen types in response to infarction. The increased *COL1A1* expression in the fetal remote tissue compared to sham could indicate fibrosis of the distal muscle from the infarct area as a result of the increased cardiac load on the surviving myocardium. The increased picrosirius red staining in the fetal MI heart tissue may be explained by the increase in *COL3A1* expression (as picrosirius red is not specific for a single collagen type while using light microscopy). Previous studies have shown that type I collagen cleavage is essential for fibrotic repair after infarction in mice (Nong et al., 2011) and that both collagen types undergo extensive remodeling after infarction (Cleutjens et al., 1995). Cardiac fibrosis is vital for immediate survival after infarction to allow scar formation and avoid the fatal occurrence of ventricular rupture (Sattler and Rosenthal, 2016). Although this scar formation allows for higher survival rates after infarction, the diminished contractile ability of the scar tissue has detrimental effects on long term heart function leading to chronic heart disease (Australian Institute of Health and Welfare [AIHW], 2015). It is clear that the fetus has a different immediate fibrotic response to infarction than an adult, however, suppression of cardiac fibrosis after infarction in large animals has not been investigated. Given that an adult human has many vascular differences compared to a fetus and small animal models [much higher blood pressure and lower heart rate (Mattson, 2001; Struijk et al., 2008)], severe consequences could be expected if scar tissue is not immediately formed. Reduced fibrosis after infarction in adults is clearly beneficial for long term chronic health outcomes, however, reduced collagen expression immediately after infarction in large animals needs to be explored further to determine if this helps or hinders acute survival in the immediate post-infarct period.

### mRNA Expression of Cell Growth Markers

Insulin-like growth factor 1 and IGF1R play a role in positively regulating cell cycle (Botting et al., 2012; Lock et al., 2018), acting indirectly upon P27 and P21, that in turn act on cyclins. Thus, the upregulation of *IGF1* in the fetal remote zone compared to sham, and lack of change in the infarct area was unexpected. One possible explanation for the lack of change or opposite mRNA expression in the fetal infarct is that the responses were governed by changes in protein turnover or by translational differences. However, a more likely explanation is the opposite expression profiles of *IGF1* and *IGF1R* may be indicative of negative feedback of this pathway. IGF expression is especially important for angiogenesis/revascularization after infarction and is associated with alpha(v) integrin activation (Dobrucki et al., 2010). It has been proposed that the neonatal and fetal mouse heart is capable of extensive vascular remodeling after infarction and this contributes to the regenerative response (Porrello et al., 2013; Aurora et al., 2014). The literature suggests IGF1 is a potent regulator of angiogenesis, our results indicate that IGF1 has a larger impact on distal tissue from the infarct and may not have as large a role in angiogenesis in a large animal model.

### Neuregulin Signaling After Infarction

Neuregulin induces cardiomyocyte proliferation through activation of the epidermal growth factor receptor (ERBB2–ERBB4) heterodimer or ERBB4–ERBB4 homodimer receptors and subsequent activation of the phosphoinositide 3-kinase (PI3K) pathway (Bersell et al., 2009). *NRG1* was increased in the adolescent animals due to infarction; this was unexpected due to neuregulin’s role in cardiomyocyte growth and proliferation. The changes in *ERBB4*, encoding a subunit of the ERBB4 receptor, may be due to reduced cardiomyocyte growth in the fetal non-infarcted tissue. The lack of change in *NRG1* expression in the fetuses also indicates that cardiomyocyte proliferation in the sheep fetus is regulated through other mechanisms than neuregulin, as several pathways act upon PI3K, such as *FGF1* and *IGF1* signaling pathways. There was a reciprocal relationship between *NRG1* and *ERBB4* after infarction between the tissue regions, which may be due to negative feedback within this pathway.

### Hippo and Meis Cell Proliferation Pathways

Cardiomyocyte proliferation can also be controlled through the transcription factor meis1, which is part of the TALE (three amino acid loop extension) family, and plays an important developmental role in embryonic cardiac morphogenesis (Mahmoud et al., 2013). *MEIS1* is associated with cell cycle arrest, and deletion of the *Meis1* gene in post-natal mouse heart causes an increase in cardiomyocyte proliferation (Mahmoud et al., 2013). The increased expression of *MEIS1* in the fetuses matched the localisation of increased proliferation present in the fetal border zone, which may indicate that the role of *MEIS1* in fetal sheep cell cycle arrest is less profound than in other animal models. YAP1, a member of the Hippo pathway, when phosphorylated stimulates cell cycle progression by interacting with transcription factors, such as TEAD (TEA domain family members), RUNX4 (RUNT-related transcription factor 4), SMAD1, T-box 5 (TBX5) and p73 (Pan, 2010; Zhao et al., 2011). Although there was an increase in *YAP1* expression in the fetal border zone, which leads to increased cell cycle progression, this data does not describe its phosphorylation status and therefore does not definitively confirm the involvement of the Hippo pathway in the immediate response to infarction. *FGF1*, much like NRG1, acts upon the PI3K pathway to activate proliferation of cardiomyocytes (Novoyatleva et al., 2014). The expression of *FGF1* confirms its central role in promoting repair in the border zone. These results indicate that any proliferation occurring in the fetal sheep heart immediately after infarction may rely more heavily on fibroblast growth factor, rather than neuregulin or insulin like growth factors to drive the PI3K pathway.

### Glucose and Fatty Acid Regulation After Infarction

Depending on the extent of ischemia, the adult heart upregulates lactate production at the expense of oxidative metabolism (Stanley, 2001; Jaswal et al., 2011). This response is logical given the restricted oxygen delivery available for fatty acid oxidation and the lack of oxygen required for glycolysis, however, this results in lactate accumulation, decreased intracellular pH and disruption of Ca2^+^ homeostasis (Stanley, 2001). Promoting oxidative glucose metabolism over lactate production may be beneficial for maintaining a favorable intracellular environment for cardiac repair, as fatty acid oxidation remains the main source of energy in the heart consuming residual oxygen (Stanley, 2001). Furthermore, during reperfusion, fatty acid oxidation also rapidly recovers, leading to an inhibition of pyruvate dehydrogenase and a further increased production of lactate and protons (Jaswal et al., 2011). SLC2A4 is the main glucose transporter in the heart after birth (Shao and Tian, 2015). The fetus has a much more limited supply of plasma glucose than the adult. Given that the fetal heart relies almost exclusively on glucose metabolism rather than fatty acid metabolism as in the adult, it is likely that the fetal heart needs to modulate the expression of glucose transporters based on glucose delivery. This change within the fetal tissue may represent an attempt to distribute metabolic resources to the infarcted area, although, the main source of glucose transport in fetal life within the heart is the basal glucose transporter SLC2A1 rather than the insulin dependent SLC2A4 (Shao and Tian, 2015).

The observed decrease in *PDK4* expression in infarcted adolescents indicates reduced mitochondrial inhibition of aerobic respiration via oxidation of pyruvate. This was an unexpected result, possibly in response to reduced glucose transport. In addition to the changes in glucose transport and metabolism, a number of interesting changes to fatty acid metabolism within infarct tissue compared to sham controls were demonstrated. Increased *FASN* expression in the adolescent heart was expected given the known reliance of adult heart tissue on fatty acid metabolism after infarction. *CPT* was also downregulated in the adolescent sheep, which is consistent with previous studies that demonstrated a downregulation in heart failure patients (Martin et al., 2000; Flanagan et al., 2010). The decreased *CPT* expression observed within the fetal remote compared to sham is due to a downregulation in the PPARγ pathway. Another possible reason for absent changes in fatty acid metabolism in the fetus could be due to the reliance on mostly glucose metabolism at this stage of development. Decreased expression of CPT in the fetal remote tissue could also be explained by increased cardiac load of the surviving myocardium since CPT has a major role in mitochondrial fatty acid metabolism rather than the preferred fetal glucose energy source. *CD36* mRNA expression was consistent with literature showing a downregulation after infarction in adult male rats (Fliegner et al., 2008). Glucose and fatty acid metabolism after infarction and may represent a crucial point for clinical intervention. The foremost differences observed between the fetal and adolescent hearts, however, could be due to the immaturity of the cardiac tissue and reliance on glucose metabolism during fetal development allowing for a greater extent of metabolic freedom.

### Technical Limitations

In the design of this study we focused on the immediate molecular and inflammatory responses to infarction. Surgery cannot be performed without anesthesia, it should be noted that although isoflurane and lignocaine have previously been used in animal studies of MI, these agents have some cardioprotective properties and may confound the clinical application of the results (Kaczmarek et al., 2009; Herdrich et al., 2010; Van Allen et al., 2012; Zgheib et al., 2014). Antibiotics were also administered to both fetuses and adolescents after surgery to prevent intrauterine infection. All these methods were standard between the Sham and MI groups to prevent any group bias. Although the study did not use a novel treatment for infarction, it is important to characterize the response to infarction, and identify/confirm important pathways before treatments targeting specific pathways can be applied. The fetal sheep has been used before to study MI (Herdrich et al., 2010; Zgheib et al., 2014). However, there are differences to these studies and the current investigation (including age of the fetal animals and regional expression analyses) that provides novel and informative data. Three days post-injury was chosen as this time-point demonstrated the largest cellular changes in previous studies in both mouse and sheep and marks the beginning of cardiomyocyte proliferation (Herdrich et al., 2010; Porrello et al., 2011; Zgheib et al., 2014; Mills et al., 2017; Quaife-Ryan et al., 2017). We therefore could not assess cardiac remodeling as this represents a chronic process, further time points would be required for assessing cardiac remodeling. Previous studies in sheep were performed in fetuses at 70 days gestation. We choose 102 days gestation when the fetus is more mature, with greater hemodynamic load but the cardiomyocytes have not yet begun to transition from mononucleated to binucleated cells. Recent evidence demonstrates that the majority of new cardiomyocytes generated during mouse heart development are cardiac progenitor cell derived (Sereti et al., 2018), the cardiomyocytes replaced after a cardiac injury, however, are mostly derived from pre-existing cardiomyocytes (Porrello et al., 2011, 2013; Haubner et al., 2012; Sereti et al., 2018). There are fewer cardiomyocytes in the cell cycle at 100 days gestation (5%) than at 70 days gestation (>10%), which may impact on the ability for regeneration to occur (Jonker et al., 2007). We therefore chose to focus our analyses on cardiomyocyte proliferation, as this is the primary driver of cardiac regeneration following injury in multiple species. Although a contribution of cardiac stem/progenitor cells cannot be ruled out, future studies will be required to determine the contribution of cardiac stem/progenitor cells to heart regeneration in fetal sheep. One possible explanation for the lack of change in mRNA expression is that the responses were governed by changes in protein turnover or by translational differences. Many studies have demonstrated that there is good agreement on the relative direction of change of protein and mRNA levels in tissue comparisons despite the lack of a precise quantitative relationship. Thus, mRNA measurements are informative of protein levels and moreover they add the considerable advantage of being able to more easily measure many mRNAs and therefore inform changes associated with many proteins. This is critical for better understanding affected regulatory pathways. There was insufficient tissue from the fetal infarcts for extensive protein expression analyses, however we, have demonstrated higher order protein expression through histological and immunohistochemical means instead, which are generally consistent with the corresponding changes in mRNA levels. In addition, Evans Blue staining was not performed in conjunction with the TTC staining as it requires perfusion of the heart tissue that would limit the subsequent analyses. Border zone tissue was therefore estimated based on TTC staining and confirmed by histological analysis. Lastly, the level of stress experienced between the two ages may be different and therefore influence results. The neonatal mouse and rat, unlike humans and sheep, are altricial, not precocial species, meaning that much of their development occurs post-natally, a result of their extremely short gestation (Morrison et al., 2018). Therefore, other than the intrauterine environment and vascular shunts in the fetal sheep model, there are less differences compared to small animal models of cardiac injury, and we anticipate the stress response to be similar.

## Conclusion

In this study we assessed the immediate (3 day) response in regenerative and quiescent hearts using a clinically relevant large animal model. This is the first study to systematically investigate inflammatory responses, fibrosis and gene expression changes during the early reparative phase in fetal and adolescent sheep. This is an important study for the field as it extends previous studies in rodents to a clinically relevant large animal model. The intra-tissue analysis used in this study is particularly important, as some changes in gene expression only occur within the border or remote zone after infarction; these changes are lost using conventional infarct vs. sham analyses. Histological and immunohistochemistry staining revealed differing localization of collagen, mature macrophage invasion and cells within cell-cycle in the MI fetuses compared with adolescent animals. Inflammatory cytokines and localisation of inflammatory cell invasion after infarction may be important for directing the regenerative and fibrotic response, along with the lineage of macrophages present in the infarcted area. Although the differences in fatty acid metabolism and glucose transport between fetuses and adolescent sheep are well understood, the response to infarction caused a dysregulation of glucose and fatty acid metabolism in the adolescent whilst the fetal hearts preserved metabolic function. Fetal hearts have a greater reliance on glycolysis with a higher percentage glucose metabolism than adults (Makinde et al., 1998). Glucose metabolism creates less CO_2_ per mol than fatty acid metabolism and also allows for anaerobic respiration when oxygen supply becomes limited (Kastendieck et al., 1988; Morrison et al., 2017). In addition, recent evidence suggests that glycolysis is required for cardiomyocyte proliferation and that fatty acid oxidation is a major contributor in blocking the cardiac cell cycle (Mills et al., 2017). This feature of the fetal heart may be beneficial in the case of local hypoxia after infarction and help reduce oxidative damage. Overall our data indicates a “resistance” to change in the fetuses that may reflect a continuation of the primary developmental plan, with altered expression profiles in the fetal remote tissue likely due to increased cardiac load on the surviving myocardium. There were opposite expression profiles observed between fetuses and adolescent sheep when comparing infarct to border and remote zone tissue. This tissue-wide response indicates a prioritization within the fetal hearts to increase proliferation/replacement of cardiomyocytes within the border zone, whilst retaining a normal developmental protocol in the infarct area. Further investigation using this model may reveal new targets for repair after MI by identifying genes and epigenetic changes with opposite expression profiles between fetal and adolescent hearts.

## Materials and Methods

### Animal Ethics and Housing

Experimental protocols for animal research were approved by the South Australian Health and Medical Research Institute (SAHMRI) Animal Ethics Committee (SAM046). Experiments were designed and reported with reference to the ARRIVE (Animal Research: Reporting of *in vivo* Experiments) guidelines (Kilkenny et al., 2010). The experiments comply with the policies and regulations of The Journal of Physiology and conform to the European Convention for the Protection of Vertebrate Animals used for Experimental and other Scientific Purposes (Grundy, 2015). We based our power calculations on previous preliminary studies to determine the number of animals required to find a statistically significant difference. Based on previous cardiomyocyte proliferation measures we expected *n* = 7 per group to yield a power of 90% with an effect size of 1.8. In total, 10 Merino ewes and their fetuses (regenerative heart) and 12 adolescent sheep (∼6 months old; non-regenerative heart) were used in this study (supplied by SAHMRI). Each ewe or adolescent sheep was housed in an individual pen in an indoor housing facility that was maintained at a constant ambient temperature of between 20–22°C with a 12 h light/dark cycle.

### Surgical Procedure to Ligate the Left Anterior Descending (LAD) Coronary Artery

At 102 days gestation (term, 150 days), ewes underwent surgery under aseptic conditions using general anesthesia induced by the intravenous infusion of diazepam (0.3 mg/kg) and ketamine (7 mg/kg) and maintained with inhalation of isoflurane (1, 2%) in oxygen. Briefly, vascular catheters (Critchley Electrical Products, Silverwater, NSW, Australia) were inserted as previously described (Danielson et al., 2005; Duan et al., 2017b) in the maternal/adolescent jugular vein, the amniotic cavity and the fetal carotid artery and jugular vein. Fetal catheters were exteriorized through a small incision in the ewe’s flank. At surgery, antibiotics were administered to the ewe/adolescent sheep intramuscularly (154 mg of Procaine penicillin, 393 mg of benzathine penicillin, 500 mg of dihydrostreptomycin; Lyppards, Adelaide, SA, Australia) and fetuses intravenously (150 mg of Procaine penicillin, 112 mg of benzathine penicillin, 250 mg of dihydrostreptomycin; Lyppards).

Animals underwent thoracotomy and were randomly allocated to sham surgery (fetus, *n* = 5; adolescent, *n* = 5) or ligation of the second diagonal of the LAD coronary artery (fetus, *n* = 7; adolescent, *n* = 7). Adolescent sheep were maintained using the same anesthesia as pregnant ewes i.e., with inhalation of isoflurane (1,2%) in oxygen. A silk suture was placed around the second diagonal of the LAD coronary artery and tied off, while observing blanching of the heart tissue. In two adolescent sheep, the procedure triggered ventricular fibrillation and these animals did not survive the surgery. From then on lignocaine was administered intravenously to all fetuses (0.2 ml bolus) and adolescents (100 mg/500 mL) prior to the permanent occlusion procedure. The thoracotomy incision was tightly sutured in layers (ribs, muscle, and skin). The fetus was returned to the uterus and the uterus was sutured closed. When the ewes and adolescent sheep were recovered from anesthesia, they were given a single dosage of analgesia (20 μg/kg, Xylazil, Troy Laboratories, Australia). Antibiotics were administered intramuscularly to each ewe or adolescent sheep for 3 days after surgery and to each fetus intra-amniotically (500 mg of ampicillin; Lyppards) for 3 days after surgery.

### Fetal Blood Gases

Fetal carotid arterial blood gas samples (0.5 mL) and adolescent jugular vein blood gas samples (1 mL) were collected for daily measurement of PO_2_, PCO_2_, pH, oxygen saturation (SO_2_), and hemoglobin (Hb) at 39°C with an ABL 520 analyzer (Radiometer, Copenhagen, Denmark) calibrated for sheep blood.

### MRI Protocol

MRI was used to measure the presence/size of the infarct immediately after the surgery and 3 days after surgery using a gadolinium chelate contrast agent (0.1 mmol/kg Gd-DTPA: injected into the fetal or adolescent jugular vein). In fetuses, we used a novel method for cardiac triggering that employs a pressure waveform transduced from a permanent indwelling vascular catheter placed in the fetal carotid artery, as recently described (Duan et al., 2017a,b). All imaging was performed on a 1.5 T Siemens Sonata scanner (Erlangen, Germany). The primary methods under investigation were single-slice inversion recovery imaging to visualize LGE, and thus the presence of infarct, and multi-slice cine myocardial imaging to quantify myocardial wall motion and ventricular volumes throughout the cardiac cycle. EGE imaging was also attempted in six fetuses (Duan et al., 2017b). EGE and LGE imaging were performed immediately after and seven minutes after injection, respectively, at a mid-ventricular short-axis slice. Cine images of the heart were obtained during the second examination only. Ten contiguous short-axis cine slices of the ventricles were obtained using a standard SSFP sequence. In fetuses, cardiac gating was achieved using a pressure waveform transduced from a permanent indwelling vascular catheter placed in the fetal carotid artery, as recently described (Duan et al., 2017a,b). In all examinations, a 3D SSFP sequence was used to image the whole fetal body for later estimation of fetal mass (Duan et al., 2017b).

### Post-processing of MRI Images

Identification of the infarcted area from the LGE and EGE images was performed qualitatively by a single experienced observer who was blinded to the subject groups. Cine images of the LV and RVs were processed separately with commercial software (QMass, MedisSuite, Netherlands) by another blinded observer. The epicardial and endocardial borders of the LV, and the endocardial border of the RV were contoured (Duan et al., 2017b). The software calculated the ESV, EDV, EF, SV, and CO of each ventricle and the EDM of the LV. These values were then indexed against the fetal and adolescent sheep weights determined at post-mortem. 3D SSFP whole fetal body images were post-processed using Mimics (Materialise, Belgium), as previously described (Duan et al., 2017a,b). The software constructed 3D models of the fetus and provided an estimation of the fetal body volume.

### Post-mortem and Tissue Collection

On the third day after ligation of the LAD and immediately after the second MRI scan was performed, ewes and adolescent sheep were humanely killed via overdose of sodium pentobarbitone (8 g; Virbac Australia, Peakhurst, NSW, Australia). The ewes’ uterus was removed by hysterotomy, and the fetus was removed and weighed. The heart was quickly dissected, weighed, and reverse perfused through the aorta with heparin sulphate (5 mL; to prevent clotting and flush blood from the heart) and a saturated KCL solution (5 mL; to arrest the heart in diastole). The heart was photographed and then cut into ∼5mm sections and the infarct was visualized using 2,3,5-triphenyltetrazolium chloride (TTC) staining. TTC is a common redox indicator used for cardiac pathology. Healthy viable heart muscle stains a bright red color by interaction with lactate dehydrogenase, while areas of potential infarctions will remain pale or unstained. Two infarcted fetuses died between surgery and post-mortem and this was detected by their blood gas measurements. A total of 10 fetuses [sham, *n* = 5 (3 Female, 2 Male); MI, *n* = 5 (3 Male, 2 Female)] and 10 adolescent sheep (sham, *n* = 5; MI, *n* = 5 all Male) underwent post-mortem and were used for molecular analyses. Ventricle tissue was collected from the Infarct area, the Border zone (salvageable tissue immediately surrounding the infarct area) and a Remote zone of the LV, as well as the corresponding areas for sham animals. Tissue was either frozen in liquid nitrogen for qRT-PCR analyses or fixed in 4% paraformaldehyde for histological or immunohistochemistry analyses.

### Real-Time PCR for Target Genes

All essential information regarding the qRT-PCR procedure are included as per the MIQE (minimum information for publication of quantitative real-time PCR experiments) guidelines (Bustin et al., 2009). Total RNA was extracted from frozen heart tissue for each fetus and adolescent sheep using QIAzol Lysis Reagent solution and QIAgen miRNeasy purification columns, as per manufacturer guidelines (Qiagen, Germany). Total RNA was quantified by spectrophotometric measurements at 260 and 280 nm in a NanoDrop Lite Spectrophotometer (Thermo Fisher Scientific). If the 260/280 nm ratio results were less than 2.1 and greater than 1.9, they were deemed acceptable for qRT-PCR. cDNA was synthesized using Superscript III First Strand Synthesis System (Invitrogen, United States) using 1 μg of total RNA, random hexamers, dNTP, DTT and Superscript III in a final volume of 20 μL, as per the manufacturer’s guidelines in a MJ Mini personal thermocycler (Bio-Rad, United States). Controls containing either no RNA transcript or no Superscript III were used to test for reagent contamination and genomic DNA contamination, respectively.

The geNorm component of qbaseplus 2.0 software (Biogazelle, Belgium) was used to determine the most stable reference genes from a panel of candidate reference genes (Vandesompele et al., 2002) and the minimum number of reference genes required to calculate a stable normalization factor, as previously described (Soo et al., 2012; McGillick et al., 2013). For qRT-PCR data output normalization, three stable reference genes *RPLP0, HPRT1, YWHAZ* (Table 3) were run in parallel with all target genes, as previously described (McGillick et al., 2014; Lock et al., 2017). A selection of genes was chosen *a priori* to investigate key pathways involved in cardiac repair as well as genes previously implicated to be involved in cardiac regeneration in small animal models (Porrello et al., 2011, 2013; Aurora et al., 2014; Zgheib et al., 2014; Han et al., 2015; Quaife-Ryan et al., 2017). Primer sets were validated and optimized as previously described (Orgeig et al., 2010; McGillick et al., 2013). Relative expression of target genes (Table 3) involved in: growth factors and cell cycle regulation (*IGF1, IGF1R, CDKN1A, CDKN1B*), extracellular matrix (*COL1A1, COL3A1*), fatty acid metabolism (*CPT, CD36, FASN, FABP*), glucose transport (*SLC2A4, PDK4)*, proliferation/cell-cycle (*MKI67, PCNA*), apoptosis and autophagy (*BCL2, BECN1*), inflammation (*TNFA, IL6, IL1β*), and pathways involved in cardiac growth and regeneration (*NRG1, ERBB4, MEIS1, YAP1, FGF1*) were measured by qRT-PCR using KiCqStart SYBR Green qPCR ReadyMix (Sigma-Aldrich, United States) in a final volume of 6 μL on a ViiA7 Fast Real-time PCR system (Applied Biosystems, United States), as previously described (McGillick et al., 2014; Lock et al., 2017). Each qRT-PCR well contained 3 μL SYBR Green Master Mix (2X), 2 μL of forward and reverse primer mixed with H_2_O to obtain final primer concentrations and 1 μL of diluted cDNA. Each sample was run in triplicate for target and reference genes. The abundance of each transcript relative to the abundance of stable reference genes (Hellemans et al., 2007) was calculated using DataAssist 3.0 analysis software (Applied Biosystems, United States) and expressed as mRNA mean normalized expression (MNE) ± SD. Outliers were defined as values that were ±2 standard deviations from the mean for each treatment group and were removed from the analysis.

**Table 3:** qRT-PCR primer information.

Primer name	Gene Symbol	Reference	Primer Sequence 5′ to 3′	Accession number
**Reference Genes**			
Ribosomal protein lateral stalk subunit P0	*RPLP0*	Passmore et al., 2009		NM_001012682.1
Hypoxanthine phosphoribosyltransferase 1	*HPRT1*	Passmore et al., 2009		NM_001034035.1
Tyrosine 3-monooxygenase	*YWHAZ*	McGillick et al., 2013		AY970970
**Growth factors and cell cycle regulators**			
Insulin-like growth factor 1	*IGF1*	Zhang et al., 2010		DQ152962
Insulin-like growth factor 1 receptor	*IGF1R*	Zhang et al., 2010		AY162434
Cyclin dependent	*CDKN1A*		F 5′ TCAGGAGGACCACTTGGA 3′	NM_001161880.1
kinase inhibitor 1A			R 5′ AATCTGTCATGCTGGTCTGG 3′	
Cyclin dependent kinase inhibitor 1B	*CDKN1B*	Lie et al., 2014		NM_001100346.1
**Neuregulin Signaling**			
Neuregulin 1	*NRG1*		F 5′ GGATAGTGGAGGATGAGGAGTA 3′	XM_012105892.2
			R 5′ GTGCCTGTGTTGTCCATTTC 3′	
Erb-b2 receptor	*ERBB4*		F 5′ CCGCCTCTCTTTCTCTTGTT 3′	XM_004004898.3
tyrosine kinase 4			R 5′ GGTTCCTCCTCTCTTTCTCTTTC 3′	
**Meis1, Hippo and FGF pathways**			
Meis homeobox 1	*MEIS1*		F 5′ AGCAGTGAGCAAGGTGATG 3′	XM_012168162.2
			R 5′ CAGAAGGGTAAGGGTGTGTTAG 3′	
Yes associated	*YAP1*		F 5′ AGCAGTTGCAGATGGAGAAA 3′	NM_001267881.2
protein 1			R 5′ TGAGACATCCCAGGAGAAGATA 3′	
Fibroblast growth	*FGF1*		F 5′ AGAACGGAAGCTCCAAACTC 3′	XM_004008910.3
factor 1			R 5′ AGCACGGCCAATGGTAAA 3′	
**Proliferation markers**			
Marker of proliferation Ki-67	*MKI67*	Orgeig et al., 2015		XM_005197116.1
Proliferating cell nuclear antigen	*PCNA*	Orgeig et al., 2015		NM_001034494.1
**Inflammatory markers**			
Tumor necrosis	*TNFA*		F 5′ ACACCATGAGCACCAAAAGC 3′	X55152
factor			R 5′ AGGCACCAGCAACTTCTGGA 3′	
Interleukin 6	*IL6*		F 5′ TCATCCTGAGAAGCCTTGAGA 3′	FJ409227
			R 5′ TTTCTGACCAGAGGAGGGAAT 3′	
Interleukin 1 beta	*IL1β*	Orgeig et al., 2015		NM_001009465.2
**Apoptosis and autophagy**			
BCL2, apoptosis regulator	*BCL2*	Botting et al., 2014		HM630309.1
Beclin 1	*BECN1*	Botting et al., 2014		XM_004012945.1
**Hypertrophy markers**			
Natriuretic	*NPPA*		F 5′ ATCACCACGAGCTTCCTCCTCTTT 3′	NM_001160027.1
peptide A			R 5′ ATACTTGTGAGGGCACAGCCTCAT 3′	
Natriuretic	*NPPB*		F 5′ CCTGCTTCTCCTCTTCTTGC 3′	NM_001160026.1
peptide B			R 5′ TAGACGGTCCAACAGCTCCT 3′	
Myosin heavy	*MYH7*		F 5′ ACCAACCTGTCCAAGTTCCG 3′	NM_174727.1
chain 7			R 5′ GCACGGCTACTCCTCATTCA 3′	
Regulator of	*RCAN1*		F 5′ CTGTCCGATGCGACCAATAA 3′	NM_001009274.1
calcineurin 1			R 5′ GAAACCTACGTATTGCCTGATCTA 3′	
ATPase	*ATP2A2*		F 5′ GATGTCGCTCCACTTCCTAATC 3′	XM_004017359.1
sarcoplasmic/endoplasmic reticulum Ca2+ transporting 2			R 5′ TCCATTAGAATCACAGGCAAGG 3′	
**Collagens**			
Collagen type I	*COL1A1*		F 5′ TTCACCTACAGCGTCACCTACGAT 3′	FJ200442
alpha 1 chain			R 5′ ATGTCGAAGCCGAATTCCTGGTCT 3′	
Collagen type III	*COL3A1*		F 5′ AACCAGAACCGTGCCAAATATGCG 3′	NM_001076831
alpha 1 chain			R 5′ TGGGCAAACTGCACAACATTCTCC 3′	
**Fatty Acid Metabolism**			
Carnitine palmitoyltransferase 1	*CPT1*	Nicholas et al., 2013		NM_001009259
Cluster of differentiation 36	*CD36*	Wang et al., 2013		BC103112.1
Fatty acid synthase	*FASN*	Lock et al., 2017; Soo et al., 2017		AF479289.1
Fatty acid binding protein	*FABP*	Zhang et al., 2016		NM_001145180.1
**Glucose transport and metabolism**			
Solute carrier family 2 member 4	*SLC2A4*	Muhlhausler et al., 2009		AB005283
Pyruvate dehydrogenase kinase 4	*PDK4*	Lie et al., 2014		XM_004007738

### Immunohistochemistry

Heart tissue sections of 5 μm thickness were cut on a Leica HistoCore manual microtome (Leica Biosystems, Germany) from one embedded fixed tissue block per animal onto SuperFrost Plus slides (VWR International, United States). Slides were baked at 60°C for 1 h followed by deparaffinization and rehydration. After rehydration, endogenous peroxide activity was blocked with 3% hydrogen peroxide (Sigma-Aldrich; United States), followed by heat-induced antigen retrieval in citrate buffer (pH 6.0). Slides were incubated overnight with the primary antibody (Ki-67 – marker of cell proliferation, or IBA1 – marker of mature macrophages) at 4°C following incubation with non-immune serum (serum blocking solution; Histostain-Plus Kit; Invitrogen, United States) to prevent nonspecific binding. Negative control slides with the primary antibody omitted were used to demonstrate no nonspecific binding of the secondary antibody or reagent contamination. In addition, negative control slides where incubation with primary antibody was substituted for rabbit serum (Sigma-Aldrich, United States) at the same protein concentration as the diluted primary antibodies were carried out at each age (Hewitt et al., 2014). Negative controls were incubated overnight at 4°C in parallel with test slides under the same experimental conditions. A Histostain-Plus kit (Invitrogen, United States) was used with horseradish peroxidase and Histostain-Plus broad spectrum 3,3′-diaminobenzidine (DAB) chromagen for visualization of positive cells. All sections were counterstained with Mayer’s hematoxylin (Sigma-Aldrich, United States). Each final antibody concentration was optimized within the immunohistochemistry protocol (as above) by manipulating a range of test conditions, as previously described (Lock et al., 2015). Antigen retrieval was carried out for all slides for each antibody. Following optimization, the substrate-chromagen reaction was allowed to occur for the same time for all slides incubated with each individual antibody. Images of stained slides at each of the ages were taken using a NanoZoomer-XR (Hamamatsu, Japan).

### Analysis of Immunohistochemistry

Ki-67 stained sections were examined using Visiopharm Computer Assisted Stereological Toolbox (NewCAST) software (Visiopharm; Hoersholm, Denmark), as previously described (Lock et al., 2015). Analysis was undertaken by a trained individual who was blinded to treatment groups. Sixty counting frames (×600 magnification) of heart tissue were randomly allocated per tissue section. Point counting using an unbiased counting frame with an area of 20,000 μm^2^ was used to estimate the numerical density of positive cells within the sections. Using the four corners of the test frame, the reference space was estimated from the number of points falling on heart tissue in each field of view. The numerical density of DAB positive cells per mm^2^ of heart tissue was obtained using the following equation (Bruel et al., 2005; McGillick et al., 2013, 2014; Lock et al., 2015):

$$DAB\;Positive\;cells\;per\;mm^{2}heart\;tissue=\frac{\sum Q^{-}\left(\mathrm{DAB}\;\mathrm{Positive}\right)}{\sum P\left(\mathrm{Heart}\;\mathrm{Tissue}\right)\times \left[\frac{a\left(frame\right)}{P}\right]}\times 10^{6}$$

where Σ*Q^-^* (DAB positive) represents the total number of DAB-positive cells counted in all counting frames of one heart tissue section, and Σ*P* (heart tissue) represents the total number of points falling on heart tissue in each field of view. *P* is the number of points that were used to count the corners included within the reference space (four corners per counting frame), and *a* is the total area of the counting frame.

### Picrosirius Red Staining and Quantification

Paraformaldehyde fixed paraffin embedded sections were sectioned at 5 μm (rotary microtome) onto superfrost slides (VWR International, United States). Slides were deparaffinized, re-hydrated and then stained using picrosirius red solution and differentiated using acid water. Slides were then dehydrated using ethanol and xylene, mounted using cytoseal resin and scanned at 40× magnification using a NanoZoomer-XR (Hamamatsu, Japan) to produce whole-slide images, which were then analyzed using the Visiomorph software (Visiopharm, Denmark) in the VIS program suite. Total area of Picrosirius Red staining was quantified as a percentage of total tissue area using custom thresholds at 20× magnification. Correct quantification of the staining was confirmed by visual examination by a trained individual who was blinded to the treatment groups.

### Statistical Analyses

We hypothesize that the immediate response to injury in (a) infarct compared with sham, and (b) infarct, border, and remote tissue, in the fetal sheep heart will be fundamentally different to the adolescent, allowing for repair after damage. Statistical analyses were performed within the GraphPad Prism Software (v7.04). All analyses were assessed for a normal distribution of data and a *P < 0.05* was considered significant. Comparison between sham and infarcted hearts using MRI and Picrosirius Red staining was by a 2-way Analysis of variance (ANOVA). KI67 Staining in the fetal hearts were assessed using a Student’s *t*-test. Gene analyses between tissue regions (Infarct vs. Border vs. Remote) at each age were by assessed using a nested ANOVA. A Bonferroni *post hoc* test was performed with multiple comparisons for each tissue region against the Sham tissue.

## Data Availability

All datasets generated for this study are included in the manuscript and/or the supplementary files.

## Author Contributions

ML, DB, EP, and JM were responsible for the conception and design of the experiments. ML, JYS, JD, SP, JBS, MS, and JM were involved in experimentation and sample/data acquisition. ML, JYS, JD, DB, SP, JBS, MS, CM, EP, RT, and JM were involved in analysis and interpretation of the data. ML and JM drafted the article. All the authors contributed to the final version.

## Conflict of Interest Statement

The authors declare that the research was conducted in the absence of any commercial or financial relationships that could be construed as a potential conflict of interest.

## Abbreviations

ATP2A2: ATPase sarcoplasmic/endoplasmic reticulum Ca2+ transporting 2
BCL2: apoptosis regulator Bcl-2
BECN1: beclin 1
CD36: cluster of differentiation 36
CDKN1A: cyclin dependent kinase inhibitor 1A
CDKN1B: cyclin dependent kinase inhibitor 1B
CO: cardiac output
COL1A1: collagen type I alpha 1 chain
COL3A1: collagen type III alpha 1 chain
CPT1: carnitine palmitoyltransferase 1
CVD: cardiovascular disease
DAB: 3,3′-diaminobenzidine
EDM: end diastolic mass
EDV: end diastolic volume
EF: ejection fraction
EGE: early gadolinium enhancement
ERBB4: Erb-b2 receptor tyrosine kinase 4
ESV: end systolic volume
FABP: fatty acid binding protein
FASN: fatty acid synthase
FGF1: fibroblast growth factor 1
HPRT1: hypoxanthine phosphoribosyltransferase 1
IGF1: insulin-like growth factor 1
IGF1R: insulin-like growth factor 1 receptor
IL1β: interleukin 1 beta
IL6: interleukin 6
LAD: left anterior descending coronary artery
LGE: late gadolinium enhancement
LV: left ventricle
MEIS1: Meis homeobox 1
MI: myocardial infarction
MKI67: marker of proliferation Ki-67
MRI: magnetic resonance Imaging
MYH7: myosin heavy chain 7
NPPA: natriuretic peptide A
NPPB: natriuretic peptide B
NRG1: neuregulin 1
PCNA: proliferating cell nuclear antigen
PDK4: pyruvate dehydrogenase kinase 4
RCAN1: regulator of calcineurin 1
RPLP0: ribosomal protein lateral stalk subunit P0
RV: right ventricle
SLC2A4: solute carrier family 2 member 4
SV: stroke volume
TNFA: tumor necrosis factor
YAP1: Yes associated protein 1
YWHAZ: tyrosine 3-monooxygenase
